# Patterns and trends of in-hospital mortality due to non-communicable diseases and injuries in Tanzania, 2006–2015

**DOI:** 10.1371/journal.pgph.0000281

**Published:** 2023-07-06

**Authors:** Leonard E. G. Mboera, Coleman Kishamawe, Susan F. Rumisha, Mercy G. Chiduo, Evord Kimario, Veneranda M. Bwana

**Affiliations:** 1 SACIDS Foundation for One Health, Sokoine University of Agriculture, Morogoro, Tanzania; 2 National Institute for Medical Research, Mwanza Research Centre, Mwanza, Tanzania; 3 National Institute for Medical Research, Headquarters, Dar es Salaam, Tanzania; 4 Malaria Atlas Project, Geospatial Health and Development, Telethon Kids Institute, West Perth, Western Australia; 5 National Institute for Medical Research, Tanga Research Centre, Tanga, Tanzania; 6 National Institute for Medical Research, Amani Research Centre, Muheza, Tanzanian; Dow University of Health Sciences, PAKISTAN

## Abstract

**Background:**

Globally, non-communicable diseases (NCD) kill about 40 million people annually, with about three-quarters of the deaths occurring in low- and middle-income countries. This study was carried out to determine the patterns, trends, and causes of in-hospital non-communicable disease (NCD) and injury deaths in Tanzania from 2006–2015.

**Methods:**

This retrospective study involved primary, secondary, tertiary, and specialized hospitals. Death statistics were extracted from inpatient department registers, death registers, and International Classification of Diseases (ICD) report forms. The ICD-10 coding system was used to assign each death to its underlying cause. The analysis determined leading causes by age, sex, annual trend and calculate hospital-based mortality rates.

**Results:**

Thirty-nine hospitals were involved in this study. A total of 247,976 deaths (all causes) were reported during the 10-year period. Of the total deaths, 67,711 (27.3%) were due to NCD and injuries. The most (53.4%) affected age group was 15–59 years. Cardio-circulatory diseases (31.9%), cancers (18.6%), chronic respiratory diseases (18.4%), and injuries (17.9%) accounted for the largest proportion (86.8%) of NCD and injuries deaths. The overall 10-year hospital-based age-standardized mortality rate (ASMR) for all NCDs and injuries was 559.9 per 100,000 population. It was higher for males (638.8/100,000) than for females (444.6/100,000). The hospital-based annual ASMR significantly increased from 11.0 in 2006 to 62.8 per 100,000 populations in 2015.

**Conclusions:**

There was a substantial increase in hospital-based ASMR due to NCDs and injuries in Tanzania from 2006 to 2015. Most of the deaths affected the productive young adult group. This burden indicates that families, communities, and the nation at large suffer from premature deaths. The government of Tanzania should invest in early detection and timely management of NCDs and injuries to reduce premature deaths. This should go hand-in-hand with continuous efforts to improve the quality of health data and its utilization.

## Introduction

Globally, non-communicable diseases (NCD) kill about 40 million people annually, with about three-quarters of the deaths occurring in low- and middle-income countries [1, 2]. The global burden of NCDs is gradually increasing with devastating health consequences in low- and middle-income countries [3]. Predictions indicate that the global burden of NCDs is expected to rise and by 2025 it will account for over 70% of all deaths [4]. The Global Burden of Disease Study 2017 already reported NCDs accounting for 73.4% of the total global deaths [5, 6]. The cumulative economic losses from NCDs are estimated to account for over 75% of the global Gross Domestic Product and are expected to surpass US$47 trillion by 2030 [7]. While there has been some progress made in improving the health of populations over the last three decades, the rise of NCDs threatens to reverse these gains and delay socio-economic development [8].

Evidence indicates an increase in the burden of NCDs in Sub-Saharan Africa [9–17]; though specific diseases and their risk factors vary considerably between countries and between urban and rural settings [17]. Studies in Nigeria, Sudan, and Tanzania have reported that NCDs account for 81% of hospital admissions among >60 years old individuals [18]. In East Africa, 40% of deaths are attributable to non-communicable diseases, which are expected to overtake communicable diseases as the leading causes of death over the next 20 years [19].

According to estimates by the World Health Organization, NCDs account for 33% of all deaths in Tanzania [20]. A recent study on causes of hospital mortality in Tanzania reported a similar level, with cardio-circulatory diseases being among the 5 leading causes [21]. Most studies in Tanzania provide data on the prevalence of diabetes, cardiovascular disease, and cancers for specific geographical areas [22–27]. A very high prevalence of hypertension among adults has been reported in both community- and hospital-based studies [23, 26, 27]. High prevalence rates of diabetes and chronic respiratory diseases have also been reported in the country [28, 29]. The rate of urbanization and unhealthy lifestyles associated with poverty and inequality have been described as the reasons for an increasing burden of NCD in Sub-Saharan Africa [17, 30]. In Tanzania, tobacco use, excessive alcohol consumption, poor diet and lack of physical activities have been reported as the most common behavioural risk factors associated with the major NCDs [32]. Cases and deaths due to road traffic injuries have increased by 44% and deaths by 64%, respectively between 1990 and 2000 [31, 32]. These factors are closely correlated with social determinant factors such as education, income, occupation, gender and ethnicity.

Although several studies have addressed the prevalence and risk factors of NCDs and injuries in Tanzania, there is limited information on the causes and mortality patterns occurring in-hospitals. In Tanzania, most often community-based data generated from national surveys have been used as sources of mortality statistics [33–37]. Nonetheless, such data are not routinely collected, only available every after 3–5 years due to high survey costs and other interferences such as pandemics and political instability, and indicators generated from these surveys may not reflect the situation in small geographical units. The causes of death are also relying on verbal autopsy methods which lack medical certification [34–39]. In-hospital mortality records are likely to be another important source of information on deaths. The cause of deaths certified by a medical practitioner is considered as the gold standard for cause-of-death reporting [38–40]. In-hospital mortality statistics derived from information reported on death certificates is among the most widely used and reliable sources of health data. However, in Sub-Saharan Africa, availability and accessibility of in-hospital deaths data where the underlying cause has been coded according to the International Classification of Diseases is low hence limit its utilization [40, 41]. This study was, therefore, carried out to determine the causes, patterns and trends of in-hospital mortality due to non-communicable diseases and injuries in Tanzania from 2006 to 2015 using data recorded at primary sources.

## Materials and methods

### Study setting and selection of study sites

This retrospective study carried out from July to December 2016 and involved 39 hospitals in Tanzania. The health system in Tanzania is structured into three functional levels: Primary level with district hospitals, secondary level with regional referral hospitals, and tertiary level with zonal referral hospitals and the national hospital (one available in Tanzania). There are also specialized hospitals that do not fit directly into the administrative levels and deal with specific conditions. The health care delivery system in Tanzania follows a cascade nature where patients access first lower facilities (dispensaries and health centres) then referred to higher levels (hospitals) when needed. Lower facilities usually serve population at small areas such as villages and wards; district and regional hospitals are assigned to serve the entire geographical areas in which they are located, while tertiary and national level hospitals serve the specific zones and the country, respectively. However, depending on accessibility, urgency of the care needed, and attraction, these facilities may serve population outside these defined areas. Most of in-hospital deaths occur in higher level facilities.

The study sites were purposively selected to ensure representation for all geographical regions in the country, epidemiological profile, and population density. Sites included one national, three zonal referral (located in 3 different zones), 20 regional referral, 11 district, and four specialised hospitals (for cancer, infectious disease specialized in tuberculosis, orthopaedic and mental health). More detail of the facilities included in this study is presented here [21].

### Data collection

Mortality data were extracted from the inpatient department (IPD) registers, death registers and International Classification of Diseases (ICD)-10 report forms available on site and filled in a customized paper-based collection tool. ICD-10 report form is a Tanzania-specific tool used to record deaths that occur in healthcare facilities with causes coded using standard ICD coding system. It is comprised of the name, age, sex, residence, date of birth, place and date of death of the deceased individual. The cause of death is usually categorised into immediate, probable, and the underlying cause. Other significant conditions and relevant medical information contributing to death are also recorded.

The extraction process was done iteratively moving from one source to another until all sources were assessed and all death events recorded were captured. The hospital staff were involved in the exercise to guide the research team all available data sources, including internal and external archives. Variables collected were deceased’s age, sex, date and cause of death. Details in data extraction have been described elsewhere [21]. This study analysed a subset of the collected data where a cause of death was mentioned as NCDs or injuries (including road traffic accidents, drowning, and animal bites).

### Statistical analysis

The ICD-10 coding system was used to assign each death to its underlying cause. Data was entered and processed in Epi-Data Software (Odense Denmark, EpiData Association, 2010) and analysed using STATA version 14 (STATA Corp LLC, Texas, USA). The hospital-based age-standardized mortality rate (ASMR) was calculated for each of the major NCDs and injuries. The death statistics were collected only from hospitals, hence the population denominator for rates calculation was adjusted to obtain the population accessing health care. The estimated rates for health care utilization for fever for Tanzania was estimated to range between 42%-75% [42]. We applied the lowest rate (42%) for year 2006 and highest (75%) in 2015 with equal increments in the years between to the annual population. No further adjustment was done to obtain population accessing care from hospitals assuming that negligible deaths occurred at lower facilities and most complicated cases are referred to higher facilities. Significant 10-year changes in trends of mortality rate for each disease, hospital type, and geographical zone were examined. A non-parametric analysis was performed, and a Mann-Kendall trend test was applied to determine presence of annual monotonic trends on the overall and cause-specific ASMR. Statistical analyses were conducted using RStudio version 1.4.1717 (RStudio Team, 2021) with the package “Kendall” and functions *prop*.*test* and *prop*.*trend*.*test* from “stats” package. Statistically significant difference was considered at 0.05 level.

### Ethics statement

This study received ethical approval from the Tanzania Medical Research Coordinating Committee of the National Institute for Medical Research (Ref. No. NIMR/HQ/R.8a/Vol. IX/2230). Extraction of data from all registers and forms ensured patient confidentiality. No individual information like names of the deceased were extracted from the sources provided; however, all entries were given identification numbers. The findings are not provided at individual level but rather in aggregated manner hence provide no link to identify individual patient data included. No informed consent was therefore required in view of the retrospective nature of this study.

## Results

A total of 247,976 deaths (all causes) were reported during the 10-year period (2006–2015) in 39 hospitals. Of the total deaths, 67,711 (27.3%) were due to non-communicable diseases and injuries. Cardio-circulatory diseases (31.9%), cancers (18.6%), chronic respiratory diseases (18.4%), and injuries (17.9%) accounted for the largest proportion of the deaths due to NCDs. The majority (56.8%) of deaths occurred among males. Males also accounted for the largest proportion of death due to injuries (78.4%; sex difference using proportion test: p-value <0.05), diabetes (58.5%), cancers (54.5%), chronic respiratory diseases (54.1%), and cardio-circulatory diseases (52.7%). Cardio-circulatory diseases were the major causes of death among both males and females (Table 1). Overall, during the 10-year period, the total deaths from NCDs increased by 153.3% from 4,298 in 2006 to 10,886 in 2015. Deaths due to cancers increased by 74.6% from 2006 to 2015, followed by diabetes (67.8%), nephritis nephrotic syndrome and nephrosis (65.7%), cardio-circulatory diseases (60.5%), injuries (62.5%), brain disorders (58.0%) and chronic respiratory diseases (47.1%).

**Table 1:** The number and percentages of cause specific NCDs and injuries by sex, 2006–2015.

Cause of death (ICD-10 Code)	Females		Males		Missing sex		Total	
	N	%	N	%	n	%	n	%
Cardio circulatory diseases (I00-I09, I11, I13, I20-I51, I60-69)	10,166	36.6	11,315	28.6	88	28.6	21,569	31.9
Cancer (C00-C97)	5,728	20.6	6,854	17.3	39	12.7	12,621	18.6
Chronic Respiratory diseases (J40-J47)	5,702	20.5	6,726	17	59	19.2	12,487	18.4
Injuries (V01-V99, W65-74, X01-X59, X60-X84, Y85-Y86)	2,602	9.4	9,452	23.9	78	25.3	12,132	17.9
Diabetes (E10-E14)	1,693	6.1	2,397	6.1	26	8.4	4,116	6.1
Brain disorders (G93-G94)	1,098	4	1,379	3.5	15	4.9	2,492	3.7
Nephritis, nephrotic syndrome and nephrosis (N00-N07, N17-N19, N25-N27)	803	2.9	1,465	3.7	3	1	2,271	3.4
Liver diseases (K72)	2	0	21	0.1	0	0	23	0
Total	27,794	100	39,609	100	308	100	67,711	100.0

The age group 15–59 years (53.4%) was the most affected category while the age group 0–4 years (4.1%) was the least affected. Over 88.4% of all deaths due to cardio-circulatory diseases affected individuals aged >15 years old; with those aged ≥60 years being most affected (pairwise proportion tests, p-value <0.05). Among children 0–4 years, the top killers were injuries and cardio circulatory diseases. While the majority of deaths due to diabetes were recorded among 15–59 years and ≥60 years old individuals, injuries, chronic respiratory diseases, cancers, and brain disorders significantly affected the 15–59 years old category (Table 2).

**Table 2:** Distribution of deaths due to non-communicable diseases by age group, 2006–2015.

Disease	0-4yrs		5-14yrs		15-59yrs		≥60yrs		Missing age		Total
	No.	%	No.	%	No.	%	No.	%	No.	%	
CCD	822	29.6	899	22.1	9,424	26.1	9,649	45.9	775	21.0	21,569
Cancer	346	12.5	586	14.4	6,787	18.8	4,293	20.4	609	16.5	12,621
CRD	44	1.6	862	21.2	7,820	21.6	2,959	14.1	802	21.8	12,487
Injury	1035	37.3	923	22.7	7,696	21.3	1,320	6.3	1,158	31.4	12,132
Diabetes	53	1.9	88	2.2	2,049	5.7	1,777	8.4	149	4.0	4,116
Brain disorders	350	12.6	624	15.4	1,148	3.2	238	1.1	132	3.6	2,492
NNN	120	4.3	83	2.0	1,203	3.3	806	3.8	59	1.6	2,271
Liver diseases	3	0.1	1	0.0	15	0.0	4	0.0	0	0.0	23
**Total**	2,773	100	4,066	100	36,142	100	21,046	100	3,684	100	67,711

**Key:** CCD = cardio-circulatory diseases; CRD = Chronic respiratory diseases; NNN = nephritis, nephrotic syndrome and nephrosis

**Fig 1:**
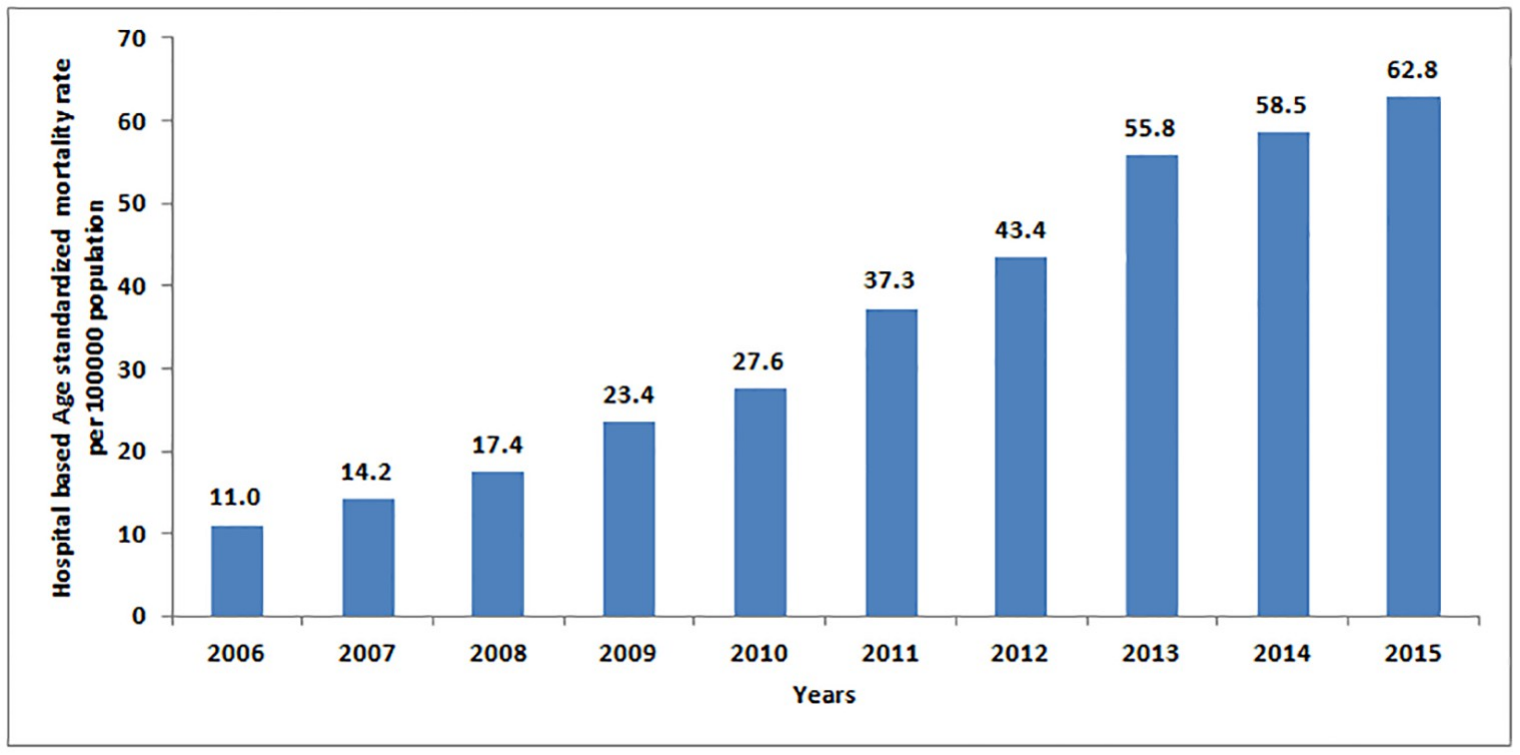
The overall age standardized mortality rate per 100,000 population, 2006–2015.

**Fig 2:**
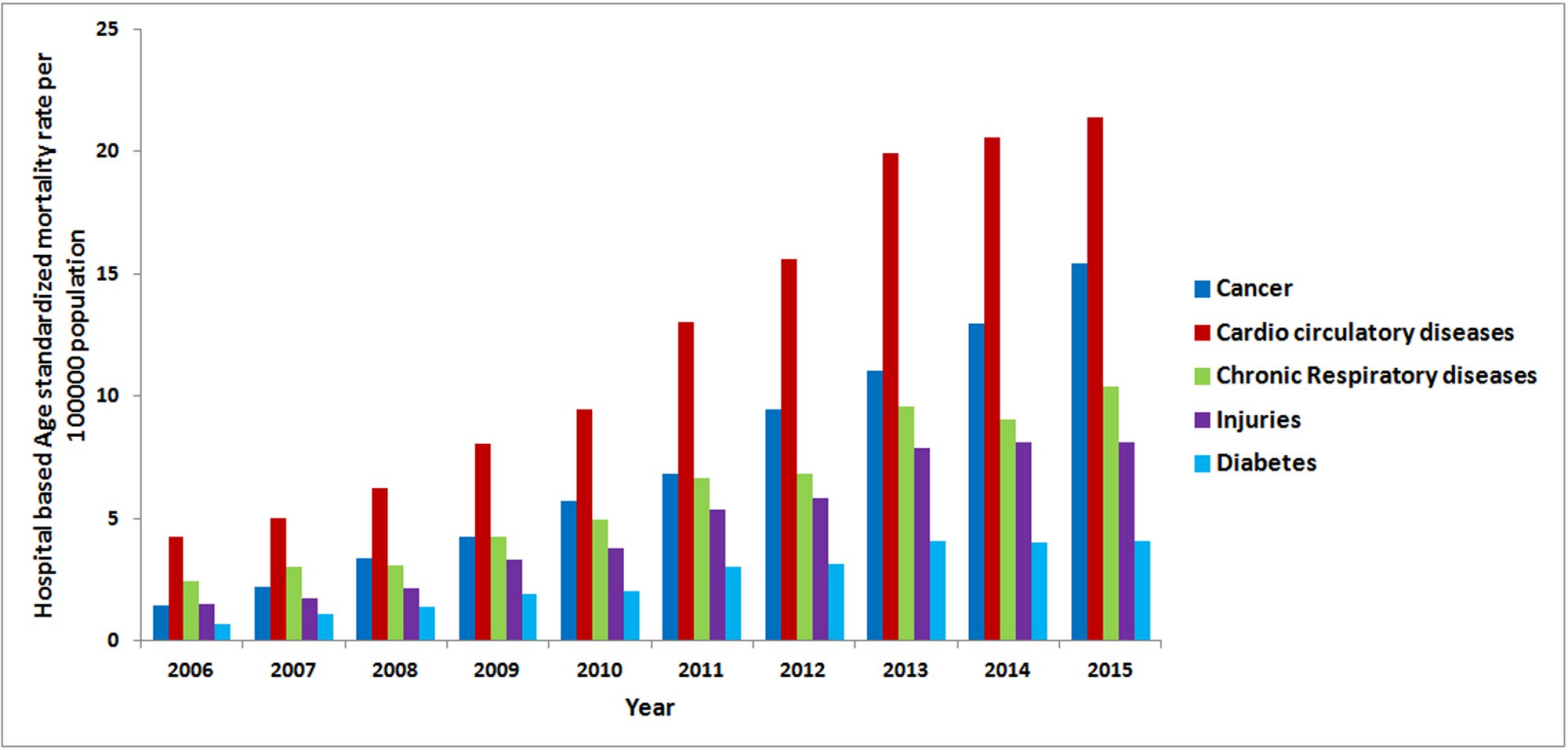
Age standardized mortality rate per 100,000 population for the top five specific NCD, 2006–2015.

The overall 10-year hospital-based age-standardized mortality rate (ASMR) for all NCDs and injuries was 559.9 per 100,000 population. It was higher among males (638.8/100,000) than females (444.6/100,000). The overall hospital-based ASMR increased from 11.0/100,000 in 2006 to 62.8/ 100,000 populations in 2015 (Fig 1, Mann–Kendall (MK) trend test: p-value <0.05). The hospital-based ASMR due to cardio-circulatory diseases increased from 4.2/100,000 in 2006 t0 21.5/100,000 in 2015. The hospital-based ASMR due to chronic respiratory diseases increased from 2.5/100,000 in 2006 to 9.6/100,000 in 2013 and remained almost stable until 2015 while that due to diabetes increased from 0.7/100,000 in 2006 to 4.1/100,000 in 2015. Similarly, hospital-based ASMR due to injuries increased from 1.5/100,000 in 2006 to 8.1/100,000 in 2015 whilst that of cancer increased from 1.5/100,000 in 2006 to 15.4/100,000 in 2015 (Fig 2). MK trend tests results for all the causes had p-value <0.05 indicating significant increasing annual trends.

Most of the deaths due to NCD occurred in the regional hospitals (40.0%) followed by zonal (27.8%), district (20.6%), and specialized hospitals (11.6%) (Table 3). While most deaths due to cancer were reported from zonal hospitals (39.9%), those due to cardio-circulatory diseases were from regional (44.2%) and zonal (31.5%) hospitals. Similarly, most deaths due to chronic respiratory diseases were reported from regional (48.1%) and district (31.1%) hospitals. Deaths due to injuries were most common in regional hospitals (44.7%).

**Table 3:** Distribution of non-communicable diseases by type of hospital, 2006–2015.

Cause of death	District		Regional		Specialized		Zonal		Total	
	No.	%	No.	%	No.	%	No.	%	No.	%
CCD	4,652	33.2	9,543	35.3	586	7.5	6,788	36.0	21,569	31.9
Cancer	786	5.6	2,322	8.6	4,482	57.2	5,031	26.7	12,621	18.6
CRD	3,889	27.8	6,010	22.2	456	5.8	2,132	11.3	12,487	18.4
Injuries	2,980	21.3	5,425	20.0	2,015	25.7	1,712	9.1	12,132	17.9
Diabetes	1,001	7.2	2,043	7.5	88	1.1	984	5.2	4,116	6.1
Brain disorders	308	2.2	944	3.5	192	2.4	1,048	5.6	2,492	3.7
NNN	338	2.4	771	2.8	23	0.3	1,139	6.0	2,271	3.4
Liver diseases	1	0.0	7	0.0	0	0.0	15	0.1	23	0.0
**Total**	13,955	100	27,065	100	7,842	100	18,849	100	67,711	**100**

**Key:** CCD = cardio-circulatory diseases; CRD = chronic respiratory diseases; NNN = nephritis, nephrotic syndrome and nephrosis

**Fig 3:**
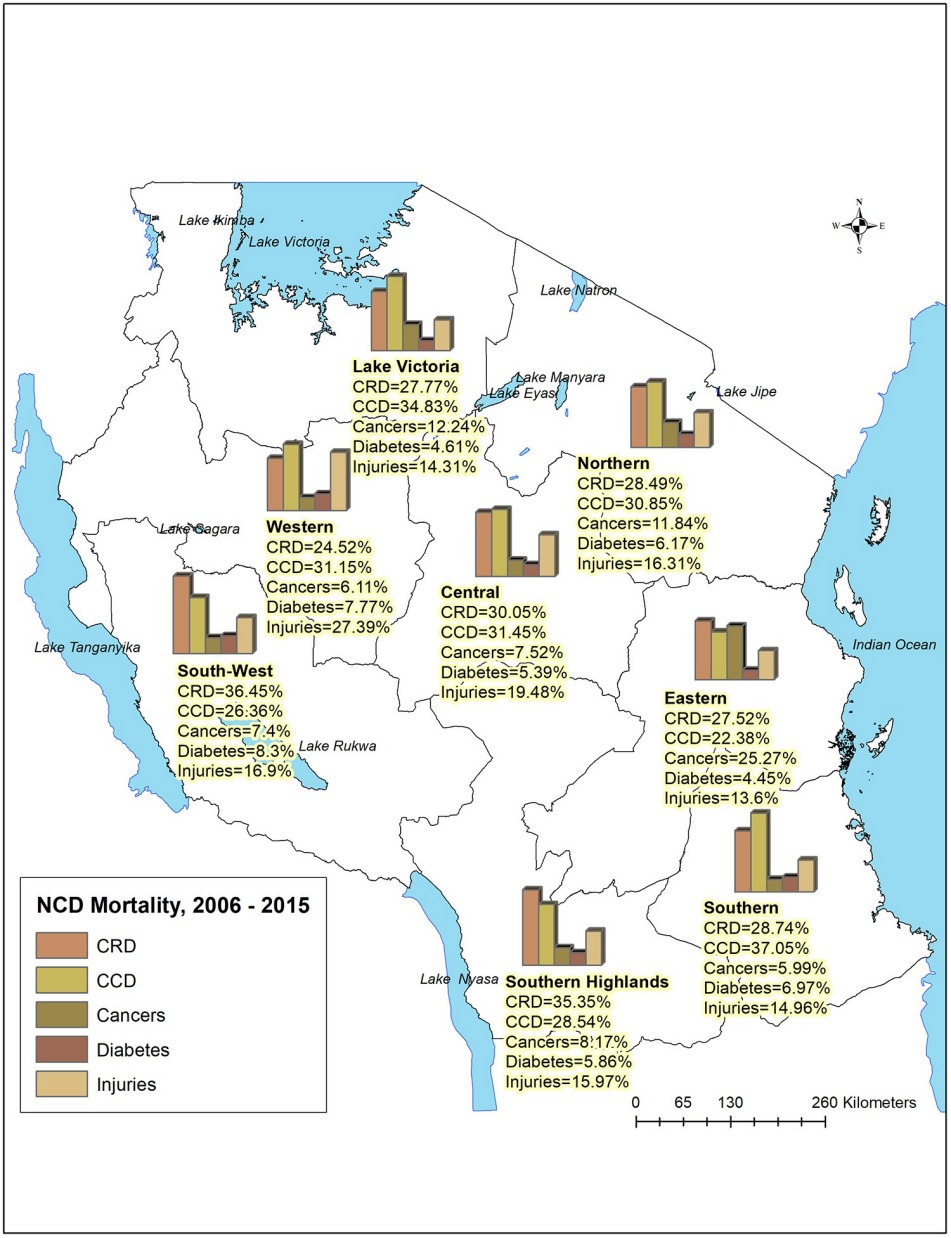
Geographical distribution of major non-communicable disease mortality in Tanzania (https://www.nbs.go.tz/index.php/en/census-surveys/gis).

There were variations in the type of non-communicable diseases by geographical distributions. Chronic respiratory diseases were the leading causes of deaths in the South-West (36.87%), Southern Highlands (35.80%), and Central Zone (30.23%). Most deaths due to cardio-circulatory diseases were reported from hospitals in the Southern (37.38%), Lake Victoria (35.20%), Central (31.64%), and Western zone (31.37%). Eastern (24.08%), Lake Victoria (11.32%), and northern (10.79%) zones reported the largest proportions of death due to cancers. Injuries accounted for the largest proportion of deaths in the Western (27.59%), Central (19.59%), and South-West zone (17.09%). Deaths due to diabetes were almost equally distributed in all zones (Fig 3).

## Discussion

This study aimed to determine the causes, patterns and trends of in-hospital mortality due to non-communicable diseases and injuries in Tanzania from 2006 to 2015. During the 10-year period (2006–2015) NCDs and injuries accounted for about one-third of all in-hospital deaths in Tanzania. The study indicates that the leading causes of death were cardio-circulatory diseases, cancers, chronic respiratory diseases and injuries. The annual hospital-based age-standardized mortality rate increased three-folds between 2006 and 2015. The rates remained high and unchanged for the last three years, with majority of deaths occurring among males and those aged 15–59 years old. Similar findings have been reported elsewhere [43, 44].

It has been described that as age increases one becomes more exposed to the risk factors for long periods until the complications develop and hence experiences the clinical syndromes of NCDs [45]. Like in our study, males accounted for most NCD deaths in studies in Brazil, Sri Lanka, and China [46–48]. Despite the observations that NCD deaths affected more males than females, several studies in Tanzania have reported that women have a higher risk of being obese than men, hence more vulnerable to several NCDs [49–51]. Overweight and obesity are major risk factors for diabetes, cardio-circulatory diseases, and cancer [52, 53]. A recent systematic review has identified several factors related to gender differences in the burden of NCDs. These include gender roles, physical access to recreational facilities, and preferences for walking and engaging in physical activity [54].

Like elsewhere in Sub-Saharan Africa, the findings of this study indicate that the contributors of NCD hospital-based mortalities, in increasing order, are cardio-circulatory diseases, cancers, chronic respiratory diseases, injuries, and diabetes [17, 20, 55, 56]. According to the World Health Organization estimates, cardio-circulatory diseases alone are responsible for 13% of the total deaths in Tanzania [4, 20]. Both cardio-circulatory diseases, cancers, and chronic respiratory diseases have been described as among the most prevalent NCDs in Tanzania [57]. Despite some of the reported trends from population-based studies, a similar trend is being observed in the hospital settings. There has been an increase in the rate of cardio-circulatory death from 9–13% between 2012 and 2016 in Tanzania [4, 57–59]. The high proportion of hospital-based cardio-circulatory deaths observed in young children may be associated with congenital malformations in neonates which include cardiac malformations and congenital heart diseases but could also be an interaction with other diseases such as HIV/AIDS, which necessitate further analysis for clarity. These children presented a significant number dying from injuries which may be linked to involvement in road traffic accidents and drowning which has been reported globally [2] which underscores the need for increased interventions on injury prevention in children. The sharp increasing trend observed during the first period of this study, 2006–2010, could be influenced by the data availability and quality [60]. However, considering strengthened information system in the later years, this study clearly indicates stagnant in the mortality rates.

The findings from this study have shown that hospital-based injuries have increased by about two-thirds during the 10-year period. The majority of the injuries in this study were caused by road traffic injuries (RTI), drowning, and animal bites. RTIs have been reported to account for the largest proportion of unintentional injuries in low-and middle-income countries [60, 61]. The overall of about 32 per 100,000 age-standardized mortality rate due to injuries in this study was similar to that reported in a population-based study in a rural district of Tanzania [32]. This may reflect improvement in health care delivery system in capturing injury-related cases or availability of modes of transferring severe injured patients to hospitals. It has already been reported that more males than females are likely to die from injuries [32]. A previous descriptive analysis of road traffic injuries in Tanzania has shown that between 1990 and 2000 the number of RTI rose by 44% and that of death by 64% [31]. Globally, each year 5.1 million people die from injuries and a quarter of these deaths are due to RTI [4] and 90% occur in low- and middle-income countries [62].

An increasing trend in hospital-based ASMR due to cancers was observed throughout the 10-year period. Recent estimates in Tanzania indicate that age-standardized in-hospital mortality rate for cancer is 47.8/100,000 population; with cervical, oesophageal, and liver cancers being among the top three causes of deaths [63]. Cancer has been described as a major emerging public health problem in Sub-Saharan Africa because of population aging and growth, as well as increased prevalence of key risk factors, including those associated with socio-economic transition [64]. Findings from this study provide additional evidence on the increasing trends in people seeking care from the conventional health system, however, may call for appropriate strategies to manage cancer cases. Diabetes was observed to be among the five leading causes of deaths due to NCDs in Tanzania with the hospital-based ASMR almost unchanged for a long period. World Health Organization’s recent statistics indicate that diabetes accounts for 1.86% of total deaths and the age-adjusted death rate is 30.24 per 100,000 populations in Tanzania [8], which is higher than the findings from this study (17.21 per 100,000). Higher population-based rate versus hospital based may indicate most deaths due to diabetes occur at home. Further research is required to identify gaps on access to appropriate care for diabetic patients which have reached severe levels. While findings of this study indicate that about half of the deaths due to diabetes which occur in hospital settings affected the 15–59 years’ age group, globally, about three-quarters of diabetes-related deaths occur in economically-productive persons under the age of 6o years [8]. Even though, the goals for clinical management are similar in older and young persons, the care for older diabetics is often complicated by their clinical and functional heterogenicity as well as comorbidities which are likely to increase the risk of dying [65].

Except for diabetes (low) and cardio-circulatory diseases (high) which presented similar contribution in almost all zones, the contributions of NCDs to the total hospital-based deaths varied by geographical zone and level of hospitals. Chronic respiratory diseases accounted for about one-third of deaths in the South-West, Southern Highlands, and Central Zones. Injuries accounted for the largest proportions of deaths in the Western, Central, and Southern Highlands. The southern zones consist of regions with cold climate which could influence high rates of chronic respiratory diseases compared to the eastern part of the country. These regions are largely rural with high levels of farming activities which may expose people to air pollution. Air pollution is the cause and risk factor of a number of chronic respiratory diseases [66]. Most injuries were due to traffic related accidents which may explain the pattern observed in the western and central zones with highway networks. Eastern, Lake Victoria, and northern zones surprisingly reported the largest proportion of deaths due to cancers. High cancer rates in these zones could be influenced by the availability of large cancer facilities (at the Ocean Road Cancer Institute, Kilimanjaro Christian Medical Centre, and Bugando Medical Centre) contributing to more people seeking care and dying in those hospitals. However, this does not necessarily translate to high cancer mortality rates among residents of those regions. These variations are likely to indicate regional and demographic differences in NCD prevalence underlying differences in lifestyle, socioeconomic status, environmental factors and access to healthcare [67]. Further studies to understand the factors behind these regional variations are necessary.

This study is likely to have several limitations. We analysed data on broadly defined disease categories such as chronic respiratory diseases, cardio-circulatory diseases, cancers, injuries, brain disorders, and kidney diseases. This limits the ability to make inferences on specific diseases. Moreover, despite the usefulness of the information that is available from this study, the hospital data are likely to provide an incomplete picture of the burden of NCDs in Tanzania, as a significant proportion of deaths occur in the community and their causes are not registered. Due to the limited scope of the study, we were not able to collect all necessary data to allow extrapolation of these deaths from the hospital-settings to population-based estimates. Data quality protocols including archiving and storage and recording practices, may affect the availability of complete data from some of the visited hospitals, thus may influence the mortality patterns and trends observed in this study. Lastly, this study used data from hospital where causes of deaths are done by health care providers, specifically physicians based on ICD-classifications. Training, routine supervision and decentralized registration systems had been done to ensure quality of medical classification of causes of death in recent years [68]. Despite these limitations, this study consolidates information on the hospital-based NCD and injuries mortality and highlights patterns and trends of major causes across Tanzania; it is our expectations that the information generated from this study will be useful to the strategies on dealing with NCD and injuries.

## Conclusions

Non-communicable diseases and injuries account for about one-third of all in-hospital deaths in Tanzania. Overall, in-hospital mortalities due to NCDs in Tanzania have increased by over half during the 10-year period. Cardio-circulatory diseases, cancers and chronic respiratory diseases are responsible for over two-thirds of the deaths and the majority of deaths affect males. The young adult age category accounts for the majority of those dying from NCDs. If the current trends continue, the probability of dying prematurely from the three main NCDs is likely to increase in Tanzania. It is important that the government of Tanzania invest in better management of NCD which includes early detection, screening, and timely treatment at all levels of the health care system. There is need to strengthen the health system that support access to preventive care and affordable medicines in order to reduce the burden of and prevent premature deaths from NCDs in Tanzania. Complementary efforts to ensure quality of health data that are used for decision making should be emphasized.
